# Prevalence and clinical significance of VHL mutations and 3p25 deletions in renal tumor subtypes

**DOI:** 10.18632/oncotarget.27428

**Published:** 2020-01-21

**Authors:** Franziska Büscheck, Christoph Fraune, Ronald Simon, Martina Kluth, Claudia Hube-Magg, Christina Möller-Koop, Imren Sarper, Kathrin Ketterer, Tjark Henke, Christian Eichelberg, Roland Dahlem, Waldemar Wilczak, Guido Sauter, Margit Fisch, Till Eichenauer, Michael Rink

**Affiliations:** ^1^Institute of Pathology, University Medical Center Hamburg-Eppendorf, Hamburg, Germany; ^2^Department of Urology, University Medical Center Hamburg-Eppendorf, Hamburg, Germany; ^3^Urologische Praxis Straubing, Straubing, Germany

**Keywords:** renal cell tumors, von Hippel-Lindau gene, VHL mutation, 3p copy number, fluorescence *in-situ* hybridization

## Abstract

Purpose: To evaluate prevalence and clinical impact of VHL mutations and deletions (3p), a cohort of consecutive kidney tumors was analyzed by DNA sequencing and fluorescence *in-situ* hybridization (FISH).

Patients and Methods: The study includes 1,805 patients with renal tumors who were surgically treated at the Department of Urology at the University Medical Center Hamburg-Eppendorf between 1994 and 2015. The cohort included 1,176 clear cell, 270 papillary, 101 chromophobe, and 28 clear cell (tubulo) papillary cancers, as well as 149 oncocytomas and 81 less common subtypes.

Results: Among 431 successfully analyzed tumors, VHL mutations were found in 59.3% of clear cell, 5.2% of papillary, 3.1% of chromophobe carcinomas and in 7.3% of oncocytomas as well as in the rare kidney tumor types (25%–60%). FISH analysis was successful in 1,403 cases. 3p25 deletion was found in 57.2% of clear cell, 17.6% of papillary, 17.7% of chromophobe carcinomas and in 11.9% of oncocytomas as well as in the rare kidney tumor types (16.7%–50%). No statistically significant associations between VHL mutation/deletion and tumor grade, stage, and clinical outcome was found. Only in the subgroup of papillary cancers, 3p deletion was significantly associated with lymph node and distant metastasis as well as with poor patient outcome (*p* < 0.05 each).

Conclusions: The presence of a VHL mutation in virtually all renal tumor subtypes suggests that VHL analysis cannot be used to distinguish between renal tumor subtypes. Consequently, anti-VHL treatment strategies should not be limited to patients with clear cell cancer.

## INTRODUCTION

The von Hippel-Lindau (VHL) tumor suppressor gene located on chromosome 3p25.3 encodes a multifunctional protein. One important function for tumor biology is serving as part of an E3 ubiquitin ligase that recognizes and ubiquitylates the α-subunits of hypoxia-induced factors (HIF-α) 1, 2 and 3, which play a crucial role for regulation of vascular formation. Inactivation of VHL mimics the consequences of hypoxia as it abrogates proteosomal HIF-α degradation, resulting in HIF-α accumulation and activation of antiapoptotic signals [1–3]. Germline mutations of VHL cause a rare familial tumor syndrome that is characterized by highly vascularized tumors. Sporadic biallelic genetic or epigenetic VHL alterations, including VHL gene mutations, chromosome 3p deletions, or VHL gene methylations, are found in the vast majority of cell renal cell carcinomas and have widely been investigated over the past years [4, 5]. Most of these studies have analyzed either mutations or deletions but rarely both. Overall the data suggest that the vast majority (> 90%) of clear cell carcinomas harbor VHL aberrations [6, 7].

Although VHL alterations are established markers of clear cell renal cell carcinomas, their role is much less clear in other kidney tumor entities. Anecdotal reports exist on VHL deletions in papillary carcinoma and clear cell (tubulo) papillary carcinoma [8–10] as well as VHL mutations in individual cases of oncocytoma as well as chromophobe and papillary cancer [11]. Regarding other less frequent tumor entities – including tumors introduced or modified by the latest WHO classification of tumors of the Urological Tract – data on VHL mutation and deletion status are also rare.

In this study, we took advantage of a cohort of more than 1,800 tumors recently reviewed according to the latest WHO classification criteria to study the combined impact of VHL mutations and deletions in kidney tumors of all types and to study the role of these alterations on clinical outcome.

## RESULTS

### Technical issues

A total of 705/1,805 (39.1%) samples of renal tumors were successfully sequenced for VHL. The successful sequencing of 3 exons was considered a prerequisite for the identification of tumors without mutations, whereas the successful sequencing of at least one altered exon was sufficient to consider tumors as positive for VHL mutations.

There were 256 (36.3%) tumors without and 449 (63.7%) tumors with VHL mutations. In 1,100 samples, an unambiguous result could not be obtained due to insufficient amount or quality of DNA (*n* = 166) or inadequate quality of the sequencing result (*n* = 934).

### VHL mutations

The frequency of VHL mutations is summarized in Table 1 for all kidney tumors and the different main histologic subtypes. Mutation rates are shown separately for tumors with sequencing data of 1–2 exons and of all 3 exons.

**Table 1:** Frequency of VHL mutations and VHL depletion of 3p in all renal cancers and in different histologic subtypes of renal cell carcinomas (RCC)

	VHL mutation analysis^*^				**3p deletion analysis**	
	**1–2 exons analyzed**		**3 exons analyzed**			
	***n***	**mutated (%)**	***n***	**mutated (%)**	***n***	**deletion (%)**
all cancers	705	63.7	431	40.6	1403	43.3
clear cell RCC	515	78.3	275	59.3	918	57.2
papillary RCC	68	19.1	58	5.2	210	17.6
oncocytoma	49	22.4	41	7.3	118	11.9
chromophobic RCC	38	18.4	32	3.1	79	17.7
clear cell (tubulo) papillary RCC	13	84.6	5	60.0	24	29.2
nephroblastoma	4	0.0	4	0.0	12	16.7
Xp11.2 translocated carcinoma	4	25.0	4	25.0	10	10.0
collecting duct carcinoma	0		0		3	0.0
metanephrogenic adenoma	0		0		3	33.3
tubular cystic RCC	1	0.0	1	0.0	2	0.0
multilocular cystic renal cell neoplasia of low malignant potential	2	50.0	2	50.0	2	50.0
acquired renal cystic disease associated RCC	1	0.0	1	0.0	1	0.0
cystic nephroma/mixed epithelial stromal tumor	1	0.0	1	0.0	1	0.0
medullary carcinoma	0		0		1	0.0
reninoma	0		0		1	100.0
n. o. s.	9	22.2	7	0.0	18	22.2

^*^analyzing less than 3 exons introduces a bias no succesful analysis for mucinar tubular spindel cell carcinoma neuroendokrine carcinoma.

The lower percentage of VHL mutations in the different tumor types with complete data sets for all 3 exons was regarded as the more “objective” real mutation rate as compared to the results of only 1–2 interpretable exons. For example, the overall VHL mutation frequency decreased from 63.7% (3 exons) to 40.6% (1–2 exons) in all cancers and dropped from 78.3% to 59.3% in clear cell carcinomas. Both chromophobe and papillary renal cell carcinomas showed comparable mutation rates of 19.1% and 18.4%, respectively, with a “corrected” value of 5.2% and 3.1%, respectively, for 3 exons. The majority of kidney tumor subtypes had – at least in some cases – VHL mutations. The number of examined tumors was very low for these four subtypes without detectable mutations: nephroblastoma (*n* = 4), tubulocystic renal cell carcinoma (*n* = 1), acquired cystic disease–associated renal cell carcinoma (*n* = 1), and cystic nephroma/mixed epithelial stromal tumor (*n* = 1). There were no associations between the mutation rate and the tumor phenotype in the two major histologic subtypes of clear cell (Table 2) and papillary renal cell carcinomas (Table 3).

**Table 2:** Association of histopathological parameters versus VHL mutations and 3p deletions in the subgroup of clear cell renal cell carcinoma

		VHL mutation analysis						3p deletion analysis		
		**1–2 exons analyzed**			**3 exons analyzed**			***n***	**del. (%)**	***P***
		***n***	**mut. (%)**	***P***	***n***	**mut. (%)**	***P***			
**ISUP**	1	123	82.9	0.3106	57	63.2	0.9009	246	61.0	0.4581
	2	185	79.5		93	59.1		302	56.6	
	3	166	74.7		98	57.1		298	54.4	
	4	33	72.7		21	57.1		64	54.7	
**Fuhrmann**	1	25	72.0	0.1269	15	53.3	0.6446	47	63.8	0.4702
	2	285	82.1		137	62.8		494	58.7	
	3	171	73.7		100	55.0		305	54.1	
	4	33	72.7		22	59.1		71	56.3	
**Thoenes**	1	162	79.6	0.1238	83	60.2	0.6302	310	60.3	0.3801
	2	290	79.7		149	60.4		489	56.0	
	3	62	67.7		42	52.4		118	54.2	
**UICC**	1	221	77.8	0.2904	120	59.2	0.3478	394	55.1	0.5170
	2	36	69.4		22	50.0		66	56.1	
	3	57	86.0		30	73.3		112	58.9	
	4	45	77.8		24	58.3		100	63.0	
**pT**	1	303	78.5	0.5410	154	57.8	0.1851	534	55.8	0.414
	2	72	73.6		37	48.6		113	55.8	
	3	130	81.5		77	68.8		252	61.9	
	4	6	66.7		5	60.0		13	53.8	
**pN**	0	66	86.4	0.4757	35	74.3	0.6521	128	55.5	0.2800
	≥1	14	78.6		9	66.7		27	66.7	
**pM**	0	67	83.6	0.4432	40	72.5	0.1983	121	58.7	0.4890
	≥1	45	77.8		23	56.5		98	63.3	

For VHL mutations, sequencing data of 1–2 exons and of all 3 exons, representing the more “objective” mutational rate are listed separately.

**Table 3:** Association of histopathological parameters versus VHL mutations and 3p deletions in the subgroup of papillary renal cell carcinoma

		VHL mutation analysis						3p deletion analysis		
		**1-2 exons analyzed**			**3 exons analyzed**			***n***	**del. (%)**	***P***
		***n***	**mut. (%)**	***P***	***n***	**mut. (%)**	***P***			
**ISUP**	1	15	20.0	0.8595	13	7.7	0.396	38	21.1	0.7056
	2	25	16.0		21	0.0		97	15.5	
	3	27	22.2		23	8.7		71	18.3	
	4	1	0.0		1	0.0		2	0.0	
**Fuhrmann**	1	1	0.0	0.7131	1	0.0	0.7989	2	0.0	0.823
	2	37	18.9		31	3.2		133	17.3	
	3	28	21.4		24	8.3		69	17.4	
	4	2	0.0		2	0.0		4	25.0	
**Thoenes**	1	13	23.1	0.4968	11	9.1	0.7264	49	18.4	0.8205
	2	52	19.2		44	4.5		146	16.4	
	3	3	0.0		3	0.0		13	23.1	
**UICC**	1	25	12.0	0.518	22	0.0	0.1528	105	17.1	0.6210
	2	10	30.0		8	12.5		22	22.7	
	3	5	20.0		5	20.0		10	10.0	
	4	6	33.3		5	20.0		14	28.6	
**pT**	1	37	16.2	0.5774	31	0.0	0.1206	138	16.7	0.2838
	2	19	26.3		16	12.5		43	16.3	
	3	7	28.6		6	16.7		19	21.1	
	4	2	0.0		2	0.0		3	66.7	
**pN**	0	8	37.5	0.2014	7	28.6	0.2817	23	0.0	0.0036
	≥1	2	0.0		2	0.0		9	33.3	
**pM**	0	7	14.3	0.6623	6	0.0	-	29	0.0	0.0028
	≥1	45	77.8		23	56.5		98	63.3	

### VHL deletions (3p25)

The frequency of 3p deletions is summarized in Table 1 for all renal tumors and for the individual histological subtypes. In addition to mutations, clear cell carcinomas also showed the highest percentage of 3p deletions with 57.2% of 918 analyzable cancers, while papillary renal cell carcinoma (17.6%), chromophobe renal cell carcinoma (17.7%) and oncocytoma (11.9%) had fewer deletions. While there was no association between 3p deletion and any unfavorable pathological parameter in clear cell carcinomas (Table 2), 3p deletions were significantly linked to the presence of lymph node and distant metastasis (<0.005 each) in papillary renal cell carcinomas (Table 3).

### Types of VHL alterations in the different renal cancer subtypes

**Figure 1:**
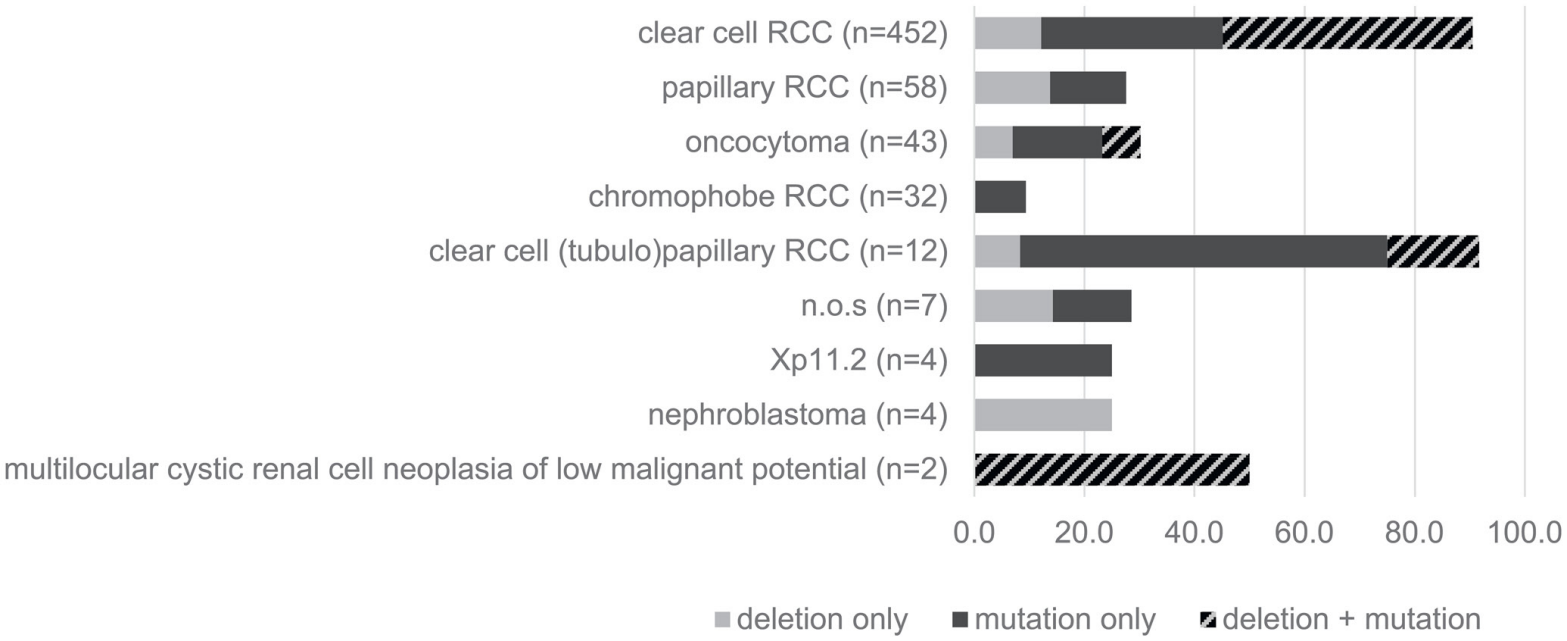
Type of VHL-alterations in different histologic subtypes of renal cell carcinomas (RCC).

**Figure 2:**
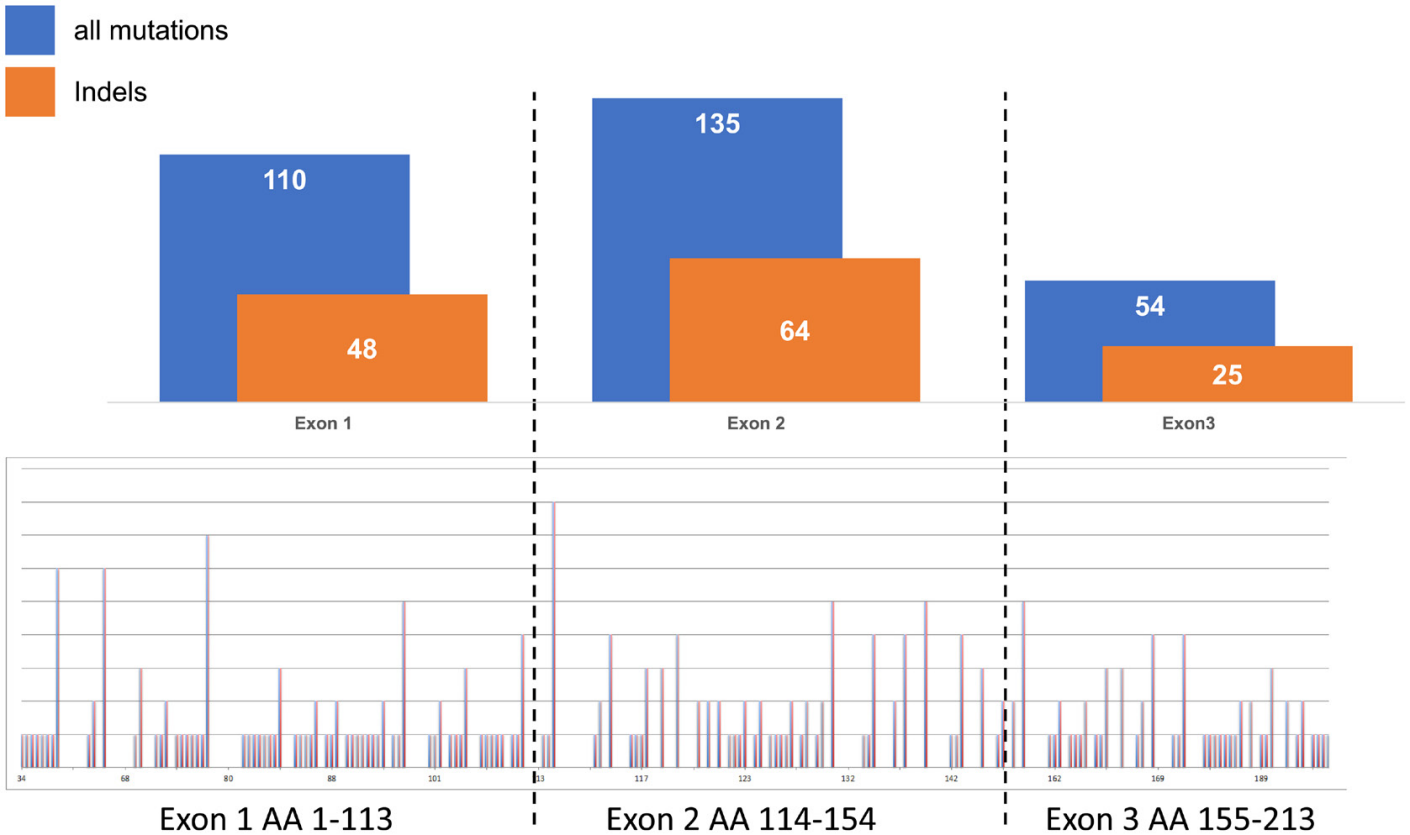
Relationship of all mutations (point and indels) versus insertions and/or deletions (indels) in exons 1–3 in clear cell renal cell carcinomas.

Highly variable ratios of VHL mutations and 3p deletions were seen in the different histologic subtypes of kidney tumors, as shown in Figure 1. Clear cell RCC (*n* = 452) not only showed highest rates of VHL alterations exceeding 80% of all cases but also high overlap of both mutation and deletion (around 50%). In contrast, VHL alterations did not exceed 30% in the other renal subtypes showing either small or no mutation/deletion overlap in oncocytoma (*n* = 43) or papillary renal cell carcinoma (*n* = 58), respectively, and a strikingly low mutation rate of approx. 10% in chromophobe renal cell carcinomas. Since sequencing analysis was not repeated for validation in cases with mutations, the number of insertions and deletions, representing the more reliable fraction of the detected mutations, were separately compared to all mutations (Figure 2, upper panel). Since their relationship was quite consistent for all 3 analyzed exons, an overall homogenous mutational pattern without hot spot regions was supported (Figure 2, lower panel).

### Association of VHL alterations with prognosis

**Figure 3:**
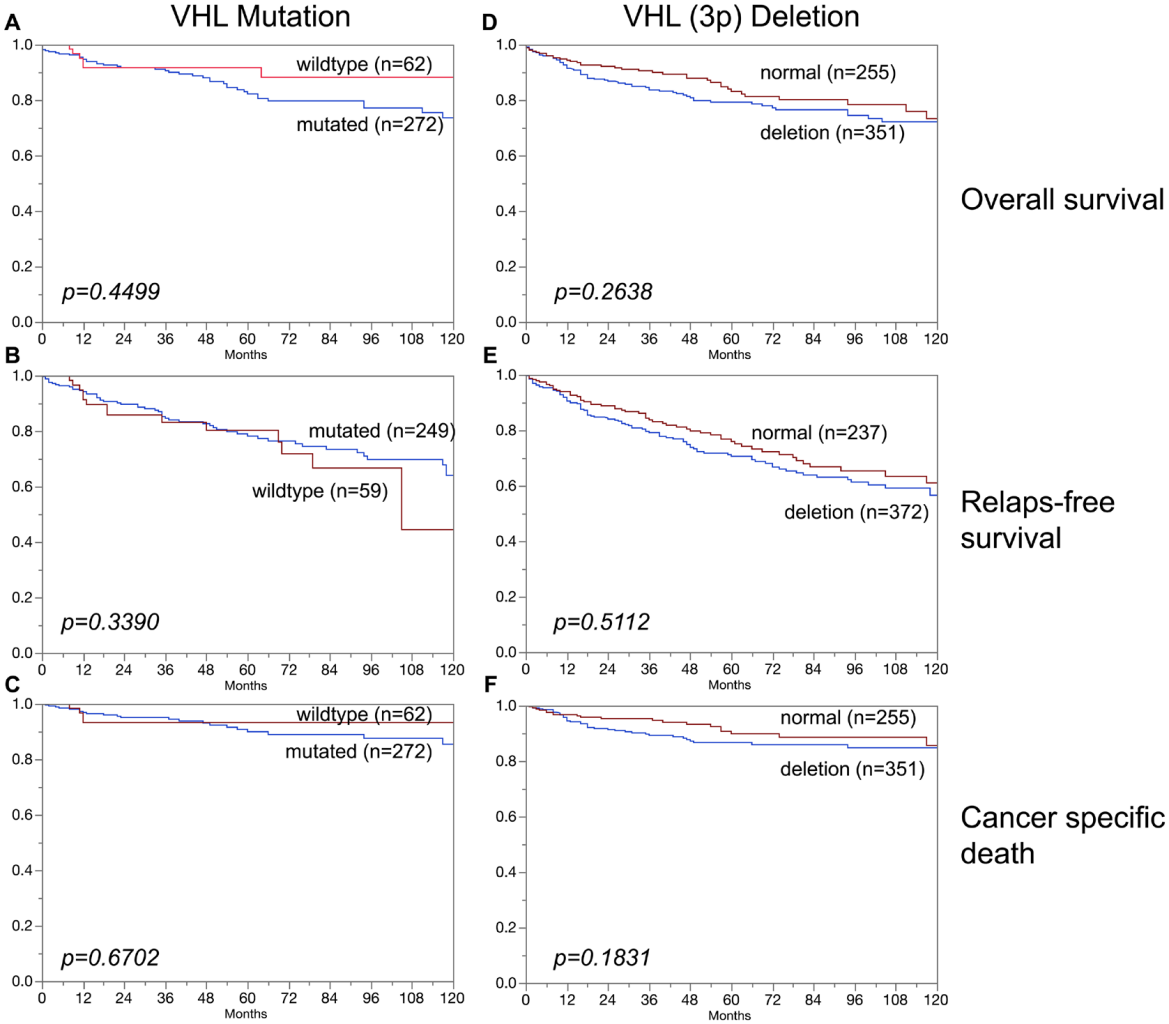
Association of (**A**–**C**) VHL mutation and (**D**–**F**) VHL deletions (3p) in clear cell carcinoma and overall survival (A, D), relapse-free survival (B, E), and cancer specific death (C, F).

**Figure 4:**
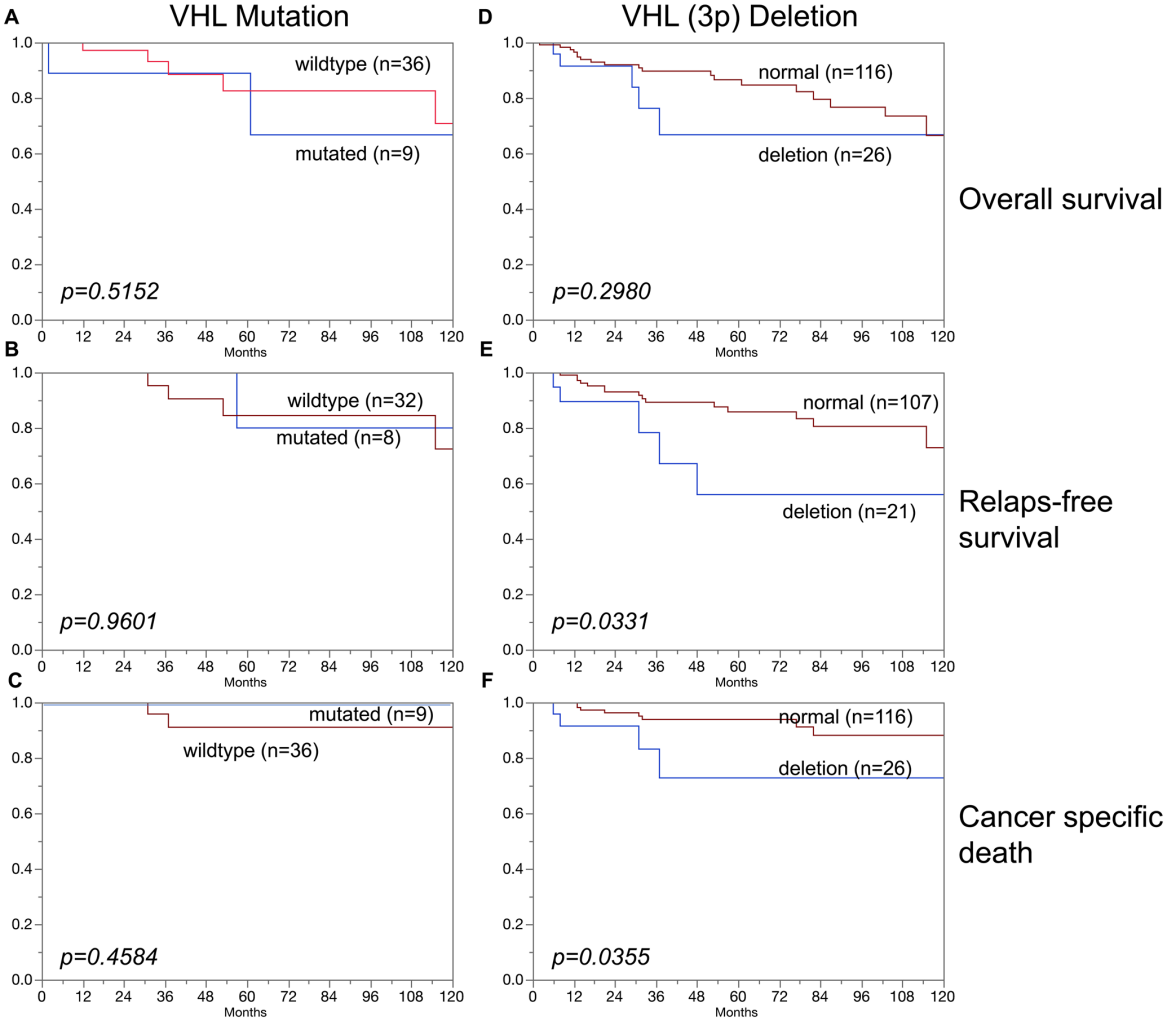
Association of (**A**–**C**) VHL mutations and (**D**–**F**) VHL deletions (3p) in papillary carcinoma and overall survival (A, D), relapse-free survival (B, E), and cancer specific death (C, F).

**Figure 5:**
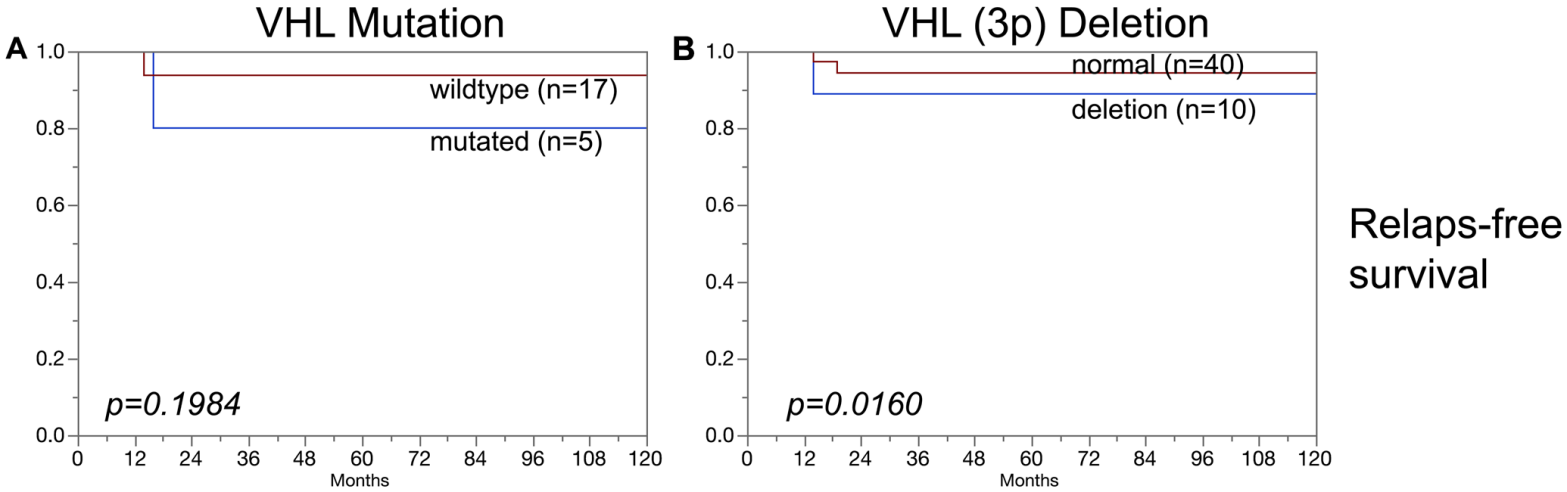
Association of (**A**) VHL mutations and (**B**) VHL deletions (3p) in chromophobe renal cell carcinomas and relapse-free survival.

In clear cell carcinoma, VHL alterations were not associated with patient prognosis regarding overall survival, relapse-free survival or cancer specific death (Figure 3A–3F). In addition, VHL mutations and 3p deletions (Figure 5B) did not show prognostic relevance for relapse-free survival in chromophobe renal cell carcinoma, based on the low number of cases. In papillary renal cell carcinoma, 3p deletions (Figure 4D–4F) but not VHL mutations (Figure 4A–4C) were significantly associated with both relapse-free survival and cancer specific death (*p* < 0.05, each).

## DISCUSSION

**Figure 6:**
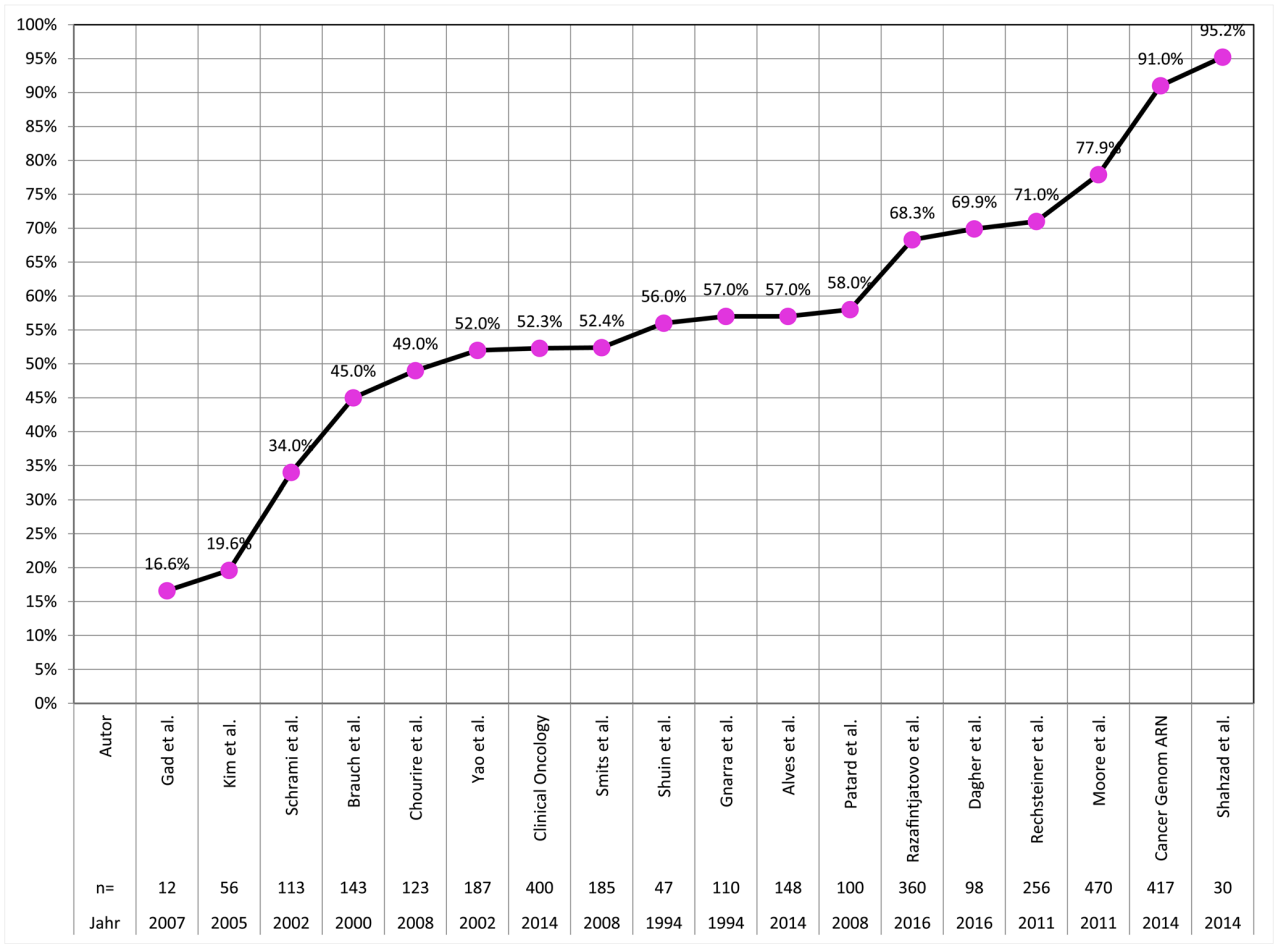
Frequency of VHL mutations in clear cell carcinoma in the literature.

A total of 1,805 formalin fixed paraffin embedded tumors collected between 1993 and 2015 were sequenced for all three VHL exons in this study. The quality of tissue (and DNA) preservation depends on the quality and duration of fixation. For example, it is necessary that the quantity of formalin exceeds the quantity of submitted tissue by a factor of 10 in transportation vessels. Because such parameters can hardly be standardized in clinical practice, it is not surprising that sequencing was not always successful. Since a mutation can be diagnosed if only one exon is successfully analyzed and this exon contains a mutation, “mutation detection” is easier than “mutation exclusion”. To exclude VHL mutation, successful analysis of all three exons is required. The mutation frequency of 59.3% found in these 275 clear cell carcinomas with complete analysis of all 3 VHL exons is likely to represent the “real” prevalence of VHL mutations in our patient cohort. This frequency ranks in the middle range of frequencies described in numerous earlier publications (Figure 6) [12–21, 4, 22–25]. Deviations between these studies might be due to cohort size or technical issues. The two studies reporting mutation rates lower than 30% studied particularly small patient cohorts (*n* = 12 and *n* = 56) [26, 12]. As in other studies, single-base point mutations were more frequent than indels, affecting several bases, and were found in all exons [4, 11].

The large number of cancers in combination with a thorough review of all tumors according to the recently published WHO criteria enabled us to collect information on VHL mutations in classical kidney tumor types and also in newly defined entities. These data demonstrate that VHL mutations predominantly occur in clear cell and clear cell (tubulo) papillary carcinoma but can – perhaps at lower frequencies – also be found in most other kidney tumor entities. In this study, we detected VHL mutations in all entities for which at least 10 cases could be successfully analyzed including 11 of 49 oncocytomas and in 7 of 38 chromophobe carcinomas (Table 1). It is of note that most others have not found VHL mutations in chromophobe carcinoma and oncocytoma [14, 27, 28], which may be explained by the low numbers of analyzed cases (*n* = 2 – 17). However, individual cases of reported VHL mutations can be found also for these entities in the literature (1 of 6 oncocytomas, 2 of 7 chromophobe carcinomas) [11]. We have validated our diagnoses of oncocytoma and/or chromophobe carcinoma in all mutated cases by histological slide review and – where necessary – by immunohistochemical analyses. Our finding of VHL mutations in papillary carcinoma matches quite well with previous reports of smaller cohorts (3/28 and 2/30 cases) [14, 11]. With respect of newly defined entities, we found VHL mutations in 1 of 2 multilocular cystic renal cell neoplasias of low malignant potential and in 1 of 4 Xp11.2 translocation carcinomas. Obviously – due to the low number of analyzed cases – we cannot exclude mutations in tubulocystic carcinoma, cystic nephroma, acquired cystic disease–associated renal cell carcinoma or nephroblastoma where we failed to find mutations in 1 to 4 analyzed specimens.

**Figure 7:**
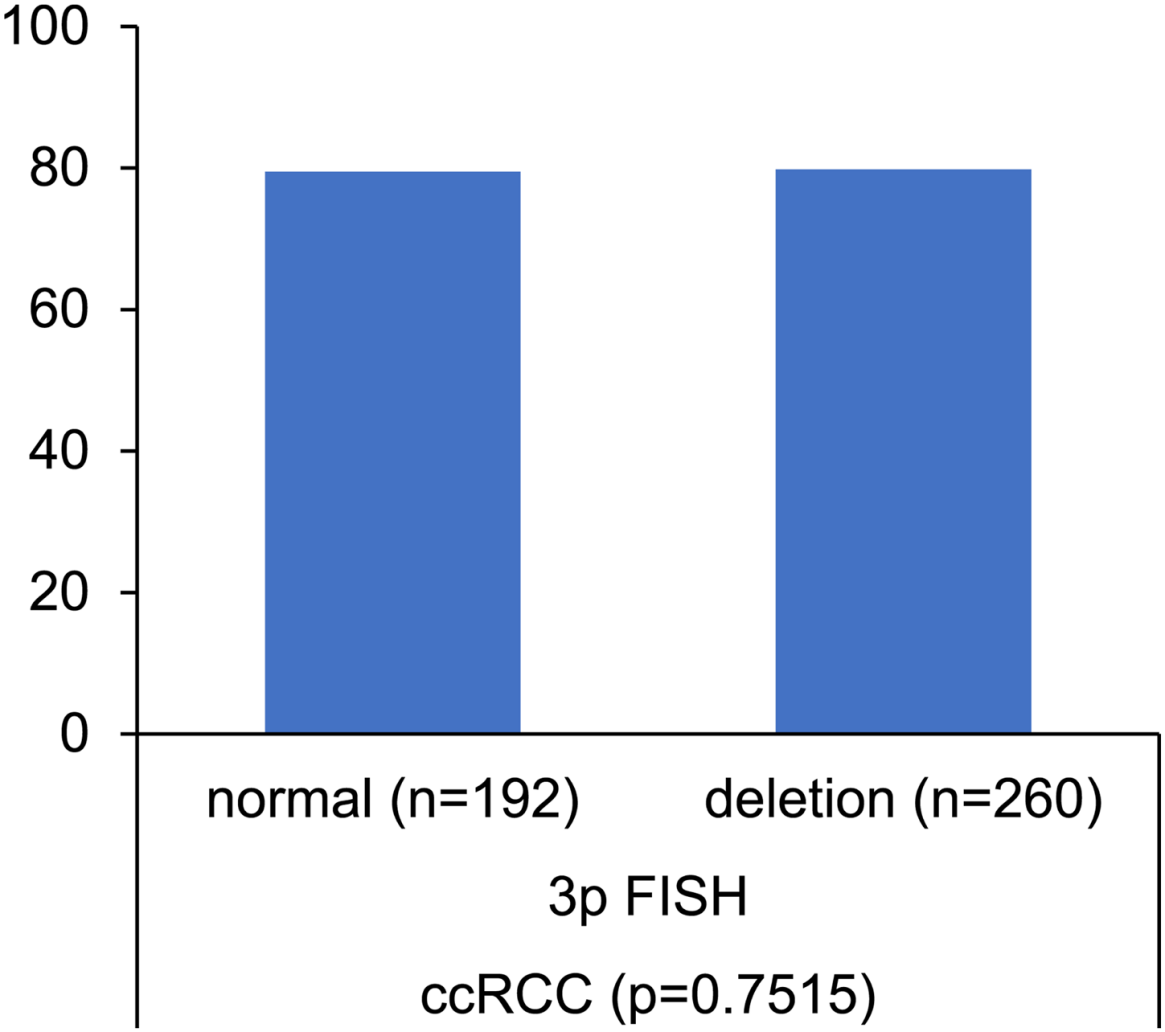
Association between VHL mutation and VHL (3p) deletion. Norm = no 3p deletion.

Deletion of the short arm of chromosome 3 (3p) is another common and well-known mechanism for VHL inactivation. It is not surprising that the distribution of 3p deletion among kidney cancer entities was comparable to the frequencies of VHL deletion. The complete lack of an association between 3p deletions and VHL mutations in our 452 clear cell carcinomas with FISH and sequencing data is unexpected, however (Figure 7). Other authors, employing LOH or CGH analysis, have earlier suggested that mutation and deletion may be statistically linked [27, 18]. The complete independency of VHL deletion and VHL mutation is consistent with a model suggesting that VHL is (almost) always completely inactivated in clear cell kidney cancer by a combination of multiple mechanisms including deletion, mutation, methylation and possibly other pathways. In such a scenario, our data suggest that there is no specific advantage for tumor cells to use the specific combination of deletion and mutation for knocking out the VHL gene.

No association was found in this study between VHL aberrations (mutation/deletion) and patient prognosis. This also hold true for the subgroups of clear cell and chromophobe carcinomas. An overwhelming majority of earlier studies had come to similar conclusions for clear cell carcinomas [29, 30]. However, one other study had suggested a prognostic role of 3p deletions in chromophobe carcinomas [31]. This is not confirmed by our data, neither for mutations nor for deletions (Figures 3, 5). It is of note that the cohorts of both Velickovic et al. (*n* = 16) and of us (*n* = 101) are too small to strongly suggest a prognostic role of VHL aberrations in chromophobe carcinomas. The cohort of analyzed papillary carcinomas was also small in our study, but evidence for a possible prognostic role of 3p deletions but not of VHL mutations for both relapse-free survival and cancer specific death arises from this analysis (Figure 4). This is supported by Velickovic et al., reporting 3p deletions in 59% of 29 papillary renal cell carcinomas significantly associated with tumor histology and patient outcome [31].

Other studies analyzing 22–37 papillary renal cell carcinoma described 3p deletions as absent or representing rare events [32–34]. Of note, the WHO/ISUP kidney cancer grading was also unrelated to presence of VHL mutations and deletions.

In summary, our data show that VHL deletions and mutations occur frequently in kidney tumors and are largely unrelated to clinical or pathological parameters. They preferentially occur in clear cell cancers but they can also be found in virtually every other kidney cancer entity. This is important because it suggests that therapies targeting the VHL pathway may not be limited to clear cell kidney cancer. It also precludes the diagnostic use of VHL aberrations for classification of kidney tumors.

## MATERIALS AND METHODS

### Patients and TMA construction

A total of 1,805 tissue specimens were available from patients with renal cell tumors, undergoing surgery between 1994–2015 at the Department of Urology, University Medical Center Hamburg-Eppendorf. Research using pseudonymized human left-over tissue samples from routine diagnosis was performed in compliance with the Helsinki Declaration, and is covered by §12a of the Hamburgisches Krankenhausgesetz (HmbKHG). Manufacturing and usage of tissue microarrays (TMAs) for research purposes has been approved by the Review Board of the Ärztekammer Hamburg (WF-049/09). The cohort mainly comprised of 1,176 (65%) patients with clear cell renal carcinoma, 270 (15%) with papillary renal cell carcinoma, 101 (6%) with chromophobe renal cell carcinoma and 149 (8%) with oncocytoma. A detailed overview of the patient cohort is given in Table 4. The mean age of the patients at date of surgery was 62 years, ranging between 1 and 94 years. Follow-up data were available from 1,171 patients with a median follow-up of 61.8 months (range: 0.3–105.4 months). Routinely formalin-fixed and paraffin embedded tissue specimens were analyzed. A tissue microarray (TMA) was constructed from all samples. The TMA manufacturing procedure was described earlier in detail [35, 36]. In brief, one 0.6 mm core per patient was taken from a representative tissue block. All 1,805 tissue cores were distributed among 4 TMA blocks, each containing 385 to 510 tumor samples.

**Table 4:** Pathological and clinical data of the arrayed renal cancers

	**Study cohort on TMA**
	**(*n* = 1,805)**
**Follow-up (months)**	
**Available (*n*)**	1,171
Mean (months)	47
Median (months)	61.8
Age (y)	
<50	267
50–70	1,011
>70	527
**Histology**	
Clear cell	1,176
Chromophobic	101
Papillary	270
Oncocytoma	149
**UICC stage**	
I	732
II	131
III	175
IV	158
**pT category**	
pT1	995
pT2	223
pT3-pT4	408
**ISUP grade**	
1	398
2	537
3	469
4	100
**Fuhrman grade**	
1	72
2	851
3	480
4	110
**Thoenes grade**	
1	497
2	839
3	177
**pN category**	
pN0	232
pN+	59
**pM category**	
pM0	220
pM+	148

Numbers do not always add up to 1,805 in the different categories because of missing data.

For VHL mutations, sequencing data of 1–2 exons and of all 3 exons, representing the more “objective” mutational rate are listed separately.

### Von Hippel-Lindau (VHL) polymerase chain reaction and sequencing

Following deparaffinization of the TMA sections for 30 min, DNA was isolated using the QIAmp DNA Micro kit (Qiagen, Hilden, Germany) according to the manufacturer’s instructions. Polymerase-chain-reaction (PCR) was performed in a 96-well plate format using 100 ng DNA in a total reaction volume of 25 µl. Three VHL-exons were amplified using the specific primers specified in Supplementary Table 1. Subsequent to PCR reaction, the service laboratory of the Eurofins-Genomics institute (Eversberg, Germany) performed sequence analysis of all PCR products using the didesoxy-method [37]. Graphical depiction and analysis of the obtained sequences were conducted using Geneious software 9.1.6 (Biomatters Ltd., New Zealand).

### 3p deletion analysis

Chromosome 3p deletion was assessed by fluorescence *in-situ* hybridization analysis (FISH) using a commercial VHL-locus specific FISH probe (Zytolight SPEC VHL/CEN 3 Dual Color Probe, ZytoVision). Prior to hybridization, 4 µm TMA sections were deparaffinized, air-dried and dehydrated in an ethanol dilution series followed by denaturation for 5 min at 74°C in 70% formamide-2xSSC solution. After overnight hybridization at 37°C in a humidified chamber, slides were washed and counterstained with 0.2 µmol/L of 4′,6-diamidino-2-phenylindole in an antifade solution. Each spot was evaluated, and the predominant signal numbers were recorded for each FISH probe. Deletion of VHL was defined as the presence of fewer VHL signals than centromere 3 probe signals in at least 60% of tumor cell nuclei.

### Statistics

Statistical calculations were performed using JMP 11 software (SAS Institute Inc., NC, USA). Contingency tables were calculated with the Chi^2^ test to search for associations between molecular parameters and tumor phenotype. Survival curves were calculated according to Kaplan-Meier. The log-Rank test was applied to detect significant differences between groups. Cox proportional hazards regression analysis was conducted to assess statistical independence and significance between clinico-pathological and molecular parameters.

## SUPPLEMENTARY MATERIALS


