# Supplementary material for: Prevalence and clinical significance of VHL mutations and 3p25 deletions in renal tumor subtypes
Source: Oncotarget. 2020 Jan 21;11(3):237–49. doi: 10.18632/oncotarget.27428 (PMC6980626; doi:10.18632/oncotarget.27428)
Supplement: Supplementary file 1 [file oncotarget-11-237-s001.pdf]

Prevalence and clinical significance of VHL mutations and 3p25 deletions in renal tumor subtypes

SUPPLEMENTARY MATERIALS

Supplementary Table 1: Listing of the specific primers directed against VHL EXON 1–3 in renal cell carcinomas (RCC)

	Pimer selection
**EXON 1**	
VHL Exon 1.1 forward	GAAGACTACGGAGGTCGA
VHL Exon 1.1 reverse	CGATTGCAGAAGATGACCT
VHL Exon 1.2 forward	GAAGAAGACGGCGGGGAG
VHL Exon 1.2 reverse	TTCAGACCGTGCTATCGT
**EXON 2**	
VHL Exon 2 forward	CGGTGTGGCTCTTTAACA
VHL Exon 2 reverse	TGTACTTACCACAACAACCT
**EXON 3**	
VHL Exon 3 forward	CCTCTTGTTCGTTCCTTGTA
VHL Exon 3 reverse	TCAGTACCATCAAAAGCTGA
